# Physics and complexity

**DOI:** 10.1098/rsta.2009.0208

**Published:** 2010-03-13

**Authors:** David Sherrington

**Affiliations:** 1 Rudolf Peierls Centre for Theoretical Physics, University of Oxford, 1 Keble Road, Oxford OX1 3NP, UK; 2 Santa Fe Institute, 1399 Hyde Park Road, Santa Fe, NM 87501, USA

**Keywords:** complex systems, spin glasses, NP-completeness, hard optimization, econophysics, neural networks

## Abstract

This paper is concerned with complex macroscopic behaviour arising in many-body systems through the combinations of competitive interactions and disorder, even with simple ingredients at the microscopic level. It attempts to indicate and illustrate the richness that has arisen, in conceptual understanding, in methodology and in application, across a large range of scientific disciplines, together with a hint of some of the further opportunities that remain to be tapped. In doing so, it takes the perspective of physics and tries to show, albeit rather briefly, how physics has contributed and been stimulated.

## 1. Introduction

Already for more than 50 years, particularly since the theoretical explanation of superconductivity by Bardeen *et al.* (1957) and as highlighted by Philip Anderson in his seminal essay ‘More is different’ (Anderson 1972), it has been recognized that the cooperative physics of interacting systems can exhibit fundamental new behaviour that is not apparent in the properties of the individual ‘elementary units’ that make up a many-body system of a very large number of these units; for example, as found in condensed matter made up of atoms, ions and electrons. Furthermore, important features of this new ‘emergent’ behaviour are often independent of the details of the make-up, for example, whether a system is fairly ‘pure’ or rather imperfect. One remarkable example is the sharp quantization of plateaux in the Hall effect of low temperature slightly disordered semiconductors (von Klitzing *et al.* 1980), providing the most accurate method of measuring the fundamental constant *e*^2^/*h*. Here, however, I shall discuss the emergence of a different phenomenon, known as ‘complexity’, concentrating on the new concepts that have arisen from its discovery and study and particularly the role of physics as a mindset and a methodology in its exploration and application.

The story has its origin in an attempt to understand the behaviour of some relatively obscure magnetic alloys that have never themselves had any technological application, but whose study has led to highly non-trivial insights of considerable subtlety, new methods of thinking, new mathematical, experimental and simulational techniques, and, in some cases, important applications, not only in many areas of physics itself, but also in information processing, hard combinatorial optimization, biology, mathematics and economics, with much further and broader potential.

As noted earlier, the systems of interest are ‘many-body’, made up of many (*N*≫1) similar individual units, with the concern the cooperative behaviour of the whole. The descriptor ‘complex’ is used to describe collective behaviour that cannot be anticipated simply from the properties of isolated individual units or from interactions among only a few of them, but arises from conflicts when large numbers of individuals have mutually incompletely satisfiable few-body rules, a feature known as ‘frustration’; indeed, complex cooperative behaviour can arise with even very simple individual units and very simple interactions. Among the consequences of this frustration and the resultant compromise are that optima and equilibria are difficult to achieve and that responses to perturbations are slow, in part extremely so, and often chaotic.

A ‘cartoon’ to illustrate the character of a complex system is of a rugged landscape with many hills and valleys and the system’s dynamics imagined as movement on the landscape with only local vision, simple moves being downward,^1^ becoming stuck in intermediate valleys and unable to surmount ridges, needing a change of rule to overcome them (for example, also allowing uphill moves), only to be faced by further barriers. In fact, the space of the landscape is very high-dimensional and the difficulty extremely much greater than for a mountain range on our three-dimensional Earth. Changing an external influence parameter, such as temperature, magnetic field or other ‘pressure’, can lead to ‘chaotic’ transformation of the whole landscape, removing the option of straightfoward iteration to a solution.

This paper is organized as follows: §2 introduces the issues and some of the concepts derived in the context of spin glasses (in condensed matter physics) and compares a related optimization problem that is simple to formulate but difficult to solve; §3 considers further hard combinatorial optimization in computer science, particularly in the context of satisfiability; §4 is devoted to some examples from biology, in the form of neural networks and proteins; §5 addresses problems involving speculative agents in finance/economics and opens the way to other social sciences; §6 considers growing networks; §7 gives some typical magnitudes; and §8 collects some conclusions and hints at the future.

## 2. The Dean’s problem and spin glasses

A simple illustration is provided by the so-called Dean’s problem in which a College Dean is faced with the task of placing students into two dormitories in such a way as to ensure that the students are as happy as possible, given that any individual pair of students might want to be in the same dormitory or in different ones.^2^ If, for any three students, the number of pairing preferences for being apart is odd, then not all these preferences can be satisfied simultaneously. This is frustration and there is no unique best choice. With a large number of students, finding the best compromise is very difficult, indeed in general NP-complete^3^ (http://www.claymath.org/millennium/P_vs_NP/).

In fact, the Dean’s problem was first considered in the context of the magnetic alloys mentioned earlier, as a minimalist model for a so-called ‘spin glass’ (Binder & Young 1986). The original spin glasses are substitutional metallic alloys such as Au_(1−*x*)_Fe_*x*_, where only the Fe ions carry magnetic moments (or ‘spins’) and, as a function of their separation, their pairwise interactions are a mixture of ferromagnetic, trying to align the two spins, and antiferromagnetic, trying to make them point in opposite directions. Experimentally (Mydosh 1995), these materials were observed to exhibit a phase transition to an unusual state, with frozen moments but no periodic order—hence, the appellation ‘glass’ by analogy with amorphous window glass, slow to respond to changes in external controls, accompanied by non-ergodicity, behaving differently depending on the order in which external perturbations, such as magnetic field or temperature, are applied. Nowadays, slow response and non-ergodicity,^4^ along with memory, ageing and rejuvenation effects, are considered the principal characteristics warranting the label ‘glass’ and the expression ‘spin glass’ is now used much more broadly to refer to systems that exhibit such glassiness owing to the combination of quenched disorder and frustration.

Both a minimal idealization of a spin glass and the Dean’s problem can be modelled with a control function (Edwards & Anderson 1975; Sherrington & Kirkpatrick 1975)
2.1
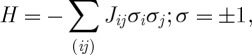

where the *i*,*j* label spins (students), *σ*=±1 indicates spin-up/down (dormitory A/B) and the interactions (preferences) {*J*_*ij*_=*J*_*ji*_} are chosen randomly and independently from a distribution *P*_*exch*_(*J*). In the case of the Dean’s problem, *H* is a cost function to be minimized. In the case of the spin glass, *H* is the Hamiltonian energy function and in thermodynamic equilibrium at a temperature *T*, the probability of a microscopic state {*σ*_*i*_} is given by
2.2
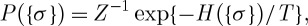

where Z is the partition function
2.3
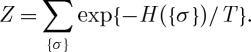

At *T*=0, the problems coincide,^5^ while equation (2.2) can also be interpreted in a Dean’s problem as the distribution resulting if the Dean allows a corresponding probabilistic degree of imprecision in his/her attempt to satisfy the students.^6^

When the temperature *T* and the mean *J* are less than critical values (proportional to the variance of *J*), there results a complex behaviour. A similar complexity is present in many other systems described by a combination of competitive interaction and disorder, including situations where the disorder is effectively self-induced through ‘sticking’ of the dynamics.

Having already noted that the Dean’s problem is NP-complete, one might reasonably ask how one knows much about the properties of these systems. One answer is through computer simulation and another is through rather subtle mathematics and physical interpretation. Also, an important point in these developments is that, in physics, one is normally interested in typical systems and their statistical properties, rather than the ‘worst case’, and the NP-hard label strictly applies to the worst-case problem. One expects the properties of a ‘good’ large many-body system to depend on the statistical distribution from which its elements are drawn, rather than the specific instances that result. This does not, however, mean that the typical case problem is trivial—indeed, the situation is very much the contrary, both technically and conceptually—and, in fact, the insight gained from typical case study has been very influential in explaining several intriguing empirical features found in several optimization problems and in predicting others, as well as in stimulating the development of new powerful techniques applicable even for specific (non-averaged) instances of hard problems. Typical behaviour study enables a powerful (and novel) set of tools to be applied and often allows the determination of the value of the best achievable average cost without the need of an actual algorithm to achieve it in specific cases.

The detailed theoretical methodologies devised to study spin glasses are beyond the scope of this article; the reader is referred instead to books such as Mézard *et al.* (1987), Young (1997) and de Dominicis & Giardina (2006). It is, however, appropriate to mention that they have involved the introduction of unusual (and, at least initially, non-rigorous) mathematics and ansätze, often guided by physical insight as well as driven pragmatically by a lack of alternatives to progress at the time. These irregular procedures led to predictions that regularly turned out to be confirmed in computer simulations of the models, as well as similar to experimental features, and provided valuable insights for new experiments with new types of probes. For the infinite-range Sherrington–Kirkpatrick (SK) spin glass,^7^ most of these results have recently been proven with rigorous mathematics.^8^ However, some subtle aspects remain controversial for spin glasses with short-range interactions (including the experimental solid-state systems that first inspired the studies). Perhaps, most importantly, however, these studies have also led to wide conceptual insights and technical applications in an enormous range of complex systems.

Despite the not inconsiderable mental and mathematical contortions that led to our current understanding, it is now possible to give physical explanation for the rugged landscape picture for the mean-field spin-glass model and its extensions. To this end, we imagine that, at the temperature of interest, our system has a number of essentially separated macrostates—let us label them by an index *S*. In macrostate *S*, the thermal average of microscopic variable *σ*_*i*_ is denoted by 〈*σ*_*i*_〉_*S*_, which, in general, can be either positive or negative and varies for different *i* or *S*. One defines the ‘overlap’ between two macrostates *S* and *S*′ as
2.4
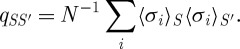

The overlap distribution is given by
2.5
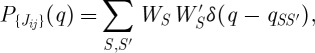

where *W*_*S*_ is the probability of macrostate *S*. In general, the macrostates depend on the specific choice of the {*J*_*ij*_}, but for the SK model, both the averaged *P*(*q*) and its higher moments can be calculated, as also other more complicated distributions of the *q*_*SS*′_, such as the correlation of pairwise overlap distributions for three macrostates *S*,*S*′ and *S*′′. The first interesting observation is that beneath a critical ‘spin-glass ordering’ temperature, the average *P*(*q*) has structure, with weight at more than one value of |*q*|, indicating that there are many non-equivalent relevant macrostates, in contrast to the situation in normal single-phase systems (paramagnetic, ferromagnetic or antiferromagnetic), where *P*(|*q*|) is a single delta function. This structure is also known as ‘replica symmetry breaking’ (RSB), following the mathematical method first employed to investigate it (Parisi 1980). Secondly, although simple physical observables, like the energy, are self-averaging, the overlap distribution is not. Thirdly, the distribution of overlaps involving three states shows that they exhibit the feature of ‘ultrametricity’, the two smallest overlaps being equal and signalling a hierarchical state structure (Mézard *et al.* 1984; Mézard & Virasoro 1985). These observations suggest a rugged landscape picture with barriers impenetrable on time scales becoming infinite with *N*. For finite-ranged spin glasses, this picture must be relaxed to have only finite barriers, but still leading to slowness on physical time scales.

*P*(*q*) cannot yet be measured directly in normal thermodynamic experiments, but it can in computer simulations (Young 1983), by running and cross-comparing identical model systems evolving separately. Also, remarkably, it turns out to be indirectly measurable in dynamics away from equilibrium (Parisi 2005), as a correction to the normal fluctuation–dissipation relation. For range-free spin glasses, one can also solve for other aspects of the dynamics away from equilibrium. This demonstrates that correlation and response functions become non-stationary in the spin-glass phase and exhibit interesting ‘ageing’ and memory effects (Cugliandolo & Kurchan 1993; Vincent 2007). Again, these evocative ‘pictures’ spread over and are also observed in other systems.

Before passing to other areas of application, it is probably worth re-emphasizing that the models leading to these understandings were *minimalist*, with as few parameters as possible; the simplest kind of spins, just two-state; the simplest type of interactions, two-spin; the simplest relevant disorder characterization, independently chosen randomly from identical distributions characterized by the minimum number of parameters. This immediately demonstrates that the rich complexity found is a consequence of the collective behaviour and not due to any complication of properties of the individual units. ‘*Complex*’ *is different from* ‘*complicated’!* Immediately, this suggests that, in other potentially complex systems, one can advantageously progress by looking for their minimal frustrated models, rather than including at the outset all aspects of real systems at the microscopic level.

Many other systems in condensed matter physics are now recognized as having conceptually related glassy complexity,^9^ but let us now turn to different subjects with different ‘rules’.

## 3. Combinatorial optimization—satisfiability

The topic of combinatorial optimization has already been introduced through the Dean’s problem, but in fact there are a very great many hard optimization problems of considerable interest and intrigue. One such class, much considered in computer science, is known as ‘satisfiability’. Here, the task is to find the values of *N* binary variables *x*_*i*_; *i*=1,…,*N*; *x*=1,0 such that a set of constraint ‘clauses’ is satisfied (SAT) e.g.3.1
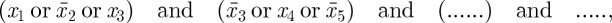
where *x*_*i*_ denotes *x*_*i*_=1 (or true) and 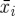 denotes *x*_*i*_=0 (or false). Here, the sections between round brackets are the clauses and the number of *x* alternatives to be satisfied within a clause is usually denoted *K* (so in the example shown, *K*=3) and the problem is referred to as K-SAT. In random K-SAT, the choices of the allocations of the *x*_*i*_ and 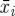 to the clauses are random (and quenched). It is a classic NP-hard problem for *K*≥3 (Kirkpatrick & Selman 1994). The interest is particularly when the number of clauses *M* scales as *M*=*αN*, with *α* independent of *N* and *N* large. The typical case becomes unsatisfiable (UNSAT) for *α* greater than a critical value *α*_c_.

Random K-SAT corresponds to an extension of the SK model with spins interacting in a combination of terms of the form *σ*_*i*_1__,…,*σ*_*i*_*p*__ where 1≤*p*≤*K* and the *i* are chosen from 1,…,*N*; this yields a graph structure that is a ‘cactus’ extension of the random graphs of Erdös & Rényi (1960).^10^ Already, therefore, one can expect analogous glassy effects in an attempt to solve the random K-SAT problem using a computer algorithm based on local operations. In fact, however, the *p*-spin version of the SK model exhibits further interesting behaviour beyond the *p*=2 SK original. In particular, there are several transitions as the temperature of this *p*-spin model is reduced. Two of these are thermodynamic; the higher, at *T*^1^_G_, is to a state known as ‘one-step replica symmetry broken’ (1RSB), corresponding to the onset of many non-equivalent macrostates {*S*} that are mutually orthogonal (Parisi 1980), followed by a lower temperature transition, at *T*^F^_G_, to ‘full replica symmetry breaking’ (FRSB) (Gardner 1985), at which the non-equivalent macrostates start to acquire a continuous range of overlaps (as characterizes the SK spin-glass phase). These transitions are, however, pre-empted by a dynamical transition at a higher temperature, *T*_D_, again to 1RSB. The fact that 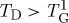 means that the system becomes glassy before the thermodynamic limit for paramagnetism is reached.

The analogue in random K-SAT is that, although, in principle, there is a limit *α*_c_ separating SAT from UNSAT, in practice, computer programs based on local dynamical algorithms are unable to reach this limit but stick at a lower *α*_*D*_, the analogue of *T*_D_. The recognition of the existence and quantification of the resultant intermediate HARD-SAT region came through the application and extension to K-SAT of statistical-physics techniques developed for spin glassses (Mézard *et al.* 2002; Mézard & Montanari 2009). In fact, the technique, known as ‘survey propagation’ (and a development of the original cavity method; Mézard *et al.* 1987), can often be applied to specific as well as averaged problems and has proven extremely useful practically.^11^

Furthermore, there are other quasi-transitions in the *p*-spin glass; for example, at a temperature higher than *T*_D_, a *p*-spin glass acquires an extensive configurational entropy that affects response and correlation functions without being thermodynamically relevant, again with an analogue in K-SAT (Krzakala *et al.* 2007). Studies of the *p*-spin glass in an applied magnetic field demonstrate that, beyond a critical field, the dynamical and thermodynamical transitions come together. One might therefore usefully consider the possibility of including an analogue of such a field in a computer algorithm to assist in the solution of hard optimization problems with effective *K*≥3, removing HARD-SAT. Complementarily, computer scientists categorize several types of NP and it is natural to look for reflections in physics.

Finally, in this section, one might note that the *p*-spin situation, with the first thermodynamic transition to 1RSB, appears to be the norm in many extensions to other spin-glass situations, particularly those characterized by a lack of symmetry between ferromagnetic and antiferromagnetic interactions, such as with Potts or quadrupolar spins, beyond critical-spin dimensionality. It is also believed to characterize the behaviour self-induced disorder in structural glasses.

## 4. Neural networks and proteins

Multi-valley landscapes are not always a nuisance—in fact, they can be very valuable. A good example is found in Hopfield’s (1982) simple model for biological neural networks where the valleys provide storage for memories and where dynamics within the valleys represents memory retrieval. In this model, one imagines the ‘brain’ as made up of a very large number of neurons, firing to different degrees, connected by a large number of ‘synapses’, both excitatory and inhibitory, the former such that a firing efferent neuron tends to make the afferent neuron fire, and the latter such that a firing efferent neuron tends to reduce the firing of the afferent neuron. If the synapses are symmetric, the collective dynamics of the neurons can be envisaged as dominantly downward motion on a high-dimensional landscape whose structure is determined by the collection of synapses. Many valleys allow for many memories, a clear requisite for a useful brain, and many valleys require frustration. That these valleys are in the high-dimensional many-neuron ‘space’ provides ‘extended memory’ and consequent robustness against failure of individual neurons or synapses.

In the spirit of equation (2.1) Hopfield idealized to *N* binary (McCulloch–Pitts) neurons (firing/non-firing; *σ*_*i*_=±1) and, in the spirit of Hebb (1949), took the synapses to be related to stored patterns through 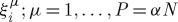 by4.1
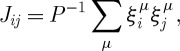
with the neural state distribution given by equation (2.2) where the ‘temperature’ *T* characterizes a sigmoidal rounding of the response of an afferent neuron’s firing to the sum of its inputs from all its many feeding efferent neurons, via their corresponding synapses (Amit 1989). The valleys then correspond to the memories 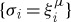 and the process of retrieval is the approach towards the minima of these valleys. Hence, there arises the concept of ‘attractors’, transcending the idealization of the modelling. Two control parameters act to separate retrieval and non-retrieval phases, the temperature *T* and the capacity *α*. The former can be viewed as ‘synaptic noise’ and the latter as ‘interference noise’ arising from the frustrating effects of competing memories. More generally, the identification of equation (4.1) is not required, as neither is the symmetry *J*_*ij*_=*J*_*ji*_, although, without this symmetry, the landscape picture of a minimizable Lyapunov function is no longer strictly true and a more subtle attractor picture is needed.

Thus far, we have imagined the interactions as ‘quenched’, fixed in time. For most practical purposes, this is true for the experimental solid-state spin glasses. However, for a neural network, it is clear that there should be learning as well as retrieval, but on a rather slower time scale. This can be achieved conceptually by permitting the synapses to evolve in response to stimuli. Indeed, as the structures of the cartoon landscapes (including the locations of valleys) are determined by the synapses, it follows that such synaptic response to external stimuli is essential to learn for future recognition and generalization—*retrieval is motion on a landscape, learning is the sculpture of the landscape*.

Other examples of systems with some frustration and with both fast and slow dynamics can be found in other aspects of biological behaviour. For example, proteins are heteropolymers with competing interactions between their amino acids and both hydrophilic and hydrophobic elements. These competitions give rise to frustration. Consequently, random heteropolymers are, in general, poor folders (in analogy with the slow dynamics and sticking of spin glasses), in contrast to proteins which must fold quickly. It has been suggested (Bryngelson & Wolynes 1987) that proteins are a special class of heteropolymers with ‘minimal frustration’, often described in cartoon terms by a ‘folding funnel’ with secondary valleys along its sides (owing to the remanent frustration). One can speculate that the proteins arose through a random process of evolution, in which, from an initial ‘soup’ of random hetero-polymers as well as subsequent mutations, the successful folders are selected through being better able to fold and hence reproduce. Similarly, it seems likely that often survival depends on unprogrammed but self-selected mutual ‘assistance’.^12^

## 5. Econophysics

More interesting examples of complex systems are found in economic and financial systems, the topic of the new science of econophysics. In these systems, the ‘units’ are people (or groups of people or institutions), speculators, producers and consumers, competing and cooperating, having different individual strategies and inclinations (e.g. of degrees of risk aversion or social conscience) and effectively interacting through information, much of it commonly available (news, internet, stock-prices, etc.). The full economic system is complicated, but an interesting minimalist model inspired by stockmarkets is provided by the ‘minority game’ (MG) (Challet *et al.* 2005). In this model, *N* ‘agents’ play a game in which, at each step, each agent makes one of the two choices (‘buy’ or ‘sell’) with the aim to make the minority choice over all agents.^13^ They make their choices based on a commonly available piece of ‘information’ but acted upon using personal strategies. In the original version, the information is the sequence of minority choices over the previous *m* time steps and each agent has two strategies, Boolean operators which, acting on the information string, yield an action decision, and the choice of which strategy to employ is determined through a personal point tally which, at each time step, rewards strategies that give the actual minority choice; in a deterministic version, each agent plays his/her strategy with the highest point tally at the time. The minority requirement corresponds to frustration, while disorder arises through quenched random choices of strategies allocated to the agents. Simulational studies of the ‘volatility’, the standard deviation of the number buying minus the number selling at each time step, show that, over many time steps, agents are effectively correlated with one another (as it differs from the value that would result from agents making independent random buy/sell choices). Rather, the volatility has a cusp minimum at a critical value of the ratio of the information dimension to the number of agents *α*=*D*/*N*=2^*m*^/*N*, suggesting a phase transition at *α*_c_. Furthermore, the system is ergodic for *α*>*α*_c_, non-ergodic for *α*<*α*_c_.^14^ Another interesting observation is that the behaviour is almost identical if the true history of the last *m* time steps is replaced by a purely fictitious ‘random’ history, provided that each agent acts as though that fictitious history were true and additionally that it spans the same information space. *Collective behaviour is determined by universal belief rather than truth!*

The MG can be studied by methods developed in the theory of spin glasses, although it should be hastened to add that there are differences. First, averaging the point-score dynamics over time scales greater than order *N* enables the elimination of the explicit information to yield a temporally coarse-grained point dynamics involving a control function of a similar structure as that of equation (2.1), but also including a random field5.1
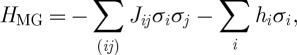
where both the *J*_*ij*_ and the *h*_*i*_ are related to the randomly chosen strategies. In a slightly different formulation of the MG, the strategies are expressible as points on the corners of a *D*-dimensional hypercube 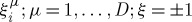. In this formulation,5.2
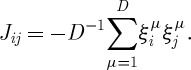
This is reminiscent of the form of the Hopfield model (equation (4.1)). However, there is an important difference in the sign, making the {*ξ*} of equation (5.2) repellors rather than attractors.

*H*_MG_ can be minimized using methods devised for spin-glass thermodynamics, but for more general behaviour, both ergodic and non-ergodic, it is necessary to use techniques developed for spin-glass macrodynamics. At least, part of this procedure is possible using a generating functional method (Coolen 2005), yielding, for large *N*, an effective single agent stochastic ensemble behaviour5.3
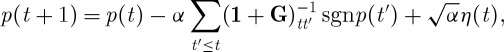
where *η*(*t*) is coloured noise determined self-consistently over the corresponding ensemble by5.4
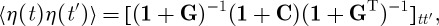
and **G** and **C** are ensemble-self-consistently determined response and correlation functions. Note that this ensemble has both memory and stochastic noise, whereas the original many-body problem had neither, and also that this mapping is valid for both ergodic and non-ergodic regimes (Galla & Sherrington 2003).^15^ *The concept of a single deterministic/rational effective agent is false, but that of an effective agent ensemble is true!*

The real world of finance and economics is significantly more complicated than the simplistic MG. However, (i) already one sees several conceptually important aspects that are likely to extend to reality; one is the inadequacy of the doctrine of ‘rational expectations’ in which it is assumed that, given the same information, every agent will make the same deduction and achieve equilibrium—it has already been pointed out that, even for the MG, there is no representative agent, but only a representative stochastic ensemble—and another is clear from the recognition that, even if agents could change their strategies, there is no optimal solution in which all have the same strategy, as, by definition, not everyone can be in the minority, (ii) the model could be extended in increments of ‘reality’ or complication, for example, including the accumulation and expenditure/gambling of varying degrees of capital, the effects of market impact, allowing for producers, who must both buy and sell in order to carry out their business, and for consumers,^16^ or involving limit-order-book bidding, settling and price movement, (iii) the inclusion of some of these effects will result in there never being any ‘equilibrium’, although there might be periods of relative stochastic quasi-steady state, albeit probably also punctuated by catastrophic crises, (iv) many of the drivers of cooperation involve information with no spatial-range restriction, thereby justifying mean-field theory treatment as a good approximation, and (v) one could investigate the introduction of global ‘friction’ and ‘bias’ mechanisms, as, for example, through the imposition of trading taxes and/or other political and legal constraints and incentives.

Indeed, one can imagine many other social-science problems involving many individuals, often grouped into intermediate local, political or national groups, where the interest is in collective behaviour under the influence of both heterogeneous inherent/cultural inclinations and national and international laws. Clearly, anticipated reactions to variation of the laws are of crucial importance in trying to anticipate the resultant ‘health’ of the whole. Minimal models and their incremental complication play an important role in separating the essential from the peripheral, in anticipating the likelihood of catrastrophes and trying to devise ‘dampers’ to avoid or minimize them. Disorder, in the guise of different inherited and culturally nurtured inclinations, such as risk aversion, honesty and emotionality, as well as mental and physical abilities, is part of the natural make-up of individuals, while frustration is a feature of their collective coexistence requiring a degree of compromise to better the whole.

## 6. Scale-free networks

Thus far, the discussion has assumed that the actual microscopic units, as well as their connectivity and/or locations, do not change. Within this restriction, several structures have been considered: crystalline/lattice (canonical experimental spin-glass alloys and the Edwards–Anderson model), fully connected (Sherrington–Kirkpatrick, *p*-spin and Hopfield models) and Erdös–Rényi graph structures (K-SAT). Simple extensions within the same general classes would include amorphous solid structures and random graphs with probabilistic occupation of bonds (Viana & Bray 1985; Fu & Anderson 1986; , as well as low-density parity check error-correcting codes (Gallagher 1963; MacKay 2003).^17^ But the last decade has seen an emergence of significant interest in another type of structure, known as scale-free (Newman *et al.* 2006; Calderelli 2007) and epitomized by many social and biological networks that have often grown spontaneously in response to need—canonical examples include the internet, hub-based airline systems, protein–protein interaction networks. In these normally random networks, the distribution of node connectivities behaves as *P*(*k*)≈*k*^−*γ*^, where *k* is the degree of connectivity (number of other nodes connected directly to the one under consideration) and *P*(*k*) is its statistical distribution. A further extension concerns ‘networks under churn’ in which nodes both enter and leave dynamically—an example is found in peer-to-peer computer networks. In these networks, varying the relative rates of entry and leaving can lead to topological changes of global structure from scale-free to exponential (Bauke & Sherrington 2007). Study of consequences of frustration and disorder in interactions of entities located on such networks is relatively in its infancy.

## 7. Magnitudes

As emphasized earlier, our interest is in systems of a large number of individuals. Some models are analytically soluble, at least in principle or in part, in limits where the number of individuals *N* is very large, 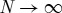, the effective number of interactions *M*=*αN* is also large and the interactions are range-free. These restrictions are rarely strictly true, but study of such systems does provide conceptual and some quantitative insight into real systems. It is perhaps worth noting some typical actual sizes. In solids, *N* is typically of the order of Avogadro’s number (*O*(10^23^)), while *α* is of the order 10 and the range is usually finite; in the brain, there are of the order 10^10^ neurons, *α* is of the order 10^5^ and the synaptic range is (relatively) long; in economics/finance, the number of interacting agents or trading units can be large, the number of effective interactions enormous^18^ and the range infinite, but effects of finiteness of *N* can be relevant;^19^ proteins vary in length from a few hundred amino acids (e.g. haemoglobin A has 287, yeasts have of the order 400–500) to the order of tens of thousands (e.g. titins in muscle).

## 8. Conclusions

In this brief perspective, I have attempted to give an impression of the conceptual, mathematical, experimental and simulational challenges and novel discoveries that the combination of disorder and frustration in many-body systems has yielded, together with hints of some of the application opportunities their recognition has offered and continues to offer. The scientific journey of discovery has been highly interdisciplinary, and there is much more scope, in many directions.

This paper has emphasized the start from and perspective of physics, but there have been other starts (and are surely other perspectives) in other subjects; for example, in evolutionary biology, the early work of Kauffman (1969, 1993) on genetic regulatory networks, employing random Boolean networks; the important work of Shannon on the achievable limit in error-correcting code theory has already been mentioned (in a footnote), but not his seminal development of the whole subject of information theory; computer scientists developed concepts like NP-hardness, a plethora of theorems, bounds and equivalences, and many methodologies including belief propagation—equivalent to mean-field theory. However, it is only in the last couple of decades that the opportunities provided by the combination of perspectives and expertises across the disciplines have been properly realized and started to be harnessed and driven.

These developments have also relied on a mutually supportive and productive interplay of three different modes of study. Two of these were previously established as methodologies, namely mathematical theory and experimentation on real systems, although the types of studies discussed above have led to many new approaches within these areas. But it has also seen the birth of and helped to drive as an equally important partner in the quest another mode of investigation that was previously little represented. This is computer simulation as an investigatory tool on idealized models, able to make observations not possible in real experiments; this is to be contrasted with the use of computers to simulate complicated ‘realistic models’ to emulate real experiments and with numerical analysis of theoretical expressions or experimental data, both of which have, of course, grown with the corresponding increase in technical computing power. For example, one can effectively perform experiments on systems whose microscopic properties are known exactly, typically corresponding to the same minimalist models as studied theoretically, without the complications of real nature and with the possibility to vary from real nature (e.g. different microscopic dynamics) and to employ probes (measurement methods) for which no analogue currently exists in the ‘real’ laboratory, but whose knowledge could check and/or guide theory. Complementarily, the need for such simplifications of ‘quasi-experimental’ systems and unphysical probes to understand complex systems has led to simulational techniques that might not otherwise have been developed, but which have proven very valuable.

There remain many further opportunities for both scientific understanding and practical application. Some can already be anticipated, but others are yet to be thought of; the developments of the last three decades have led to so many remarkable and unanticipated discoveries that it seems inevitable that many more will arise. The next few decades offer the prospect of much richness for the scientific explorer and technological applier alike.

## Footnotes

1 Physicists usually think in terms of cost functions to minimize, while evolutionary biologists think in terms of complementary fitnesses to maximize. I shall use the physics convention; the mapping between them is just a minus sign.

2 For simplicity, we shall assume that feelings between pairs of students are mutual.

3 Non-deterministic polynomial (NP) is the class of problems where we can verify, but not necessarily find, a solution in time polynomial in the number of elements *N*. NP-complete problems are the hardest ones in NP, and are believed to take exponential time to solve (Garey & Johnson 1979). Note that in the Dean’s problem, the total number of possible choices is 2^*N*^ and we are interested in large *N*.

4 An ergodic system equilibrates and its behaviour under the application of more than one perturbation is independent of the order in which those perturbations are applied. In a non-ergodic system, there is no complete equilibration and the order of application matters; a standard example for a spin glass is the combination of application of a magnetic field and of a change of temperature; beneath a critical spin-glass temperature, the susceptibility measured by first cooling and then applying a field (‘field-cooled’ susceptibility) is greater than obtained by the opposite order of events (zero-field-cooled susceptibility).

5 More precisely, the Dean’s problem corresponds to the SK spin glass where the sum in equation (2.1) is over all (*ij*) and each *J*_*ij*_(=*J*_*ji*_) is drawn independently from the same distribution. To model a short-range spin glass, the {*i*,*j*} are chosen as neighbours on a lattice of appropriate dimensionality; three for normal experience.

6 In fact, introduction of an analogue of temperature has proven to be a very valuable tool in optimization, using the so-called ‘simulated annealing’ (Kirkpatrick *et al.* 1983), in which stochastic imprecision is deliberately introduced into a computer updating algorithm in order to stochastically overcome barriers in a simple energy/cost description, and is then gradually reduced.

7 Also known as the mean-field spin glass.

8 So far, all the results obtained rigorously for infinite-range spin glasses (Talagrand 2006) have been in accordance with the earlier less rigorously deduced results arising from Parisi’s ansatz (Parisi 1980).

9 Although the spins may be pseudo-descriptions of other physical attributes (e.g. Wang *et al.* 2007; Sherrington 2008).

10 A further convenient graphical description employs factor graphs with two kinds of vertices corresponding, respectively, to the *x* and to their interaction groupings (Mézard & Montanari 2009).

11 It is useful when small loops in the graph stucture are not important.

12 For example, autocatalytic sets (Kauffman 1995).

13 The philosophy is to emulate the idea that one gets the best price by selling when most people want to buy or buying when most people want to sell.

14 As characterized by the irrelevance or relevance of a bias in the initial point scores.

15 The many-body problem could be generalized to include memory and stochasticity, but the point here is that they are not necessary to produce them in the effective single-agent ensemble.

16 Often referred to as ‘liquidity providers’, the financial analogue of energy input, such as provided by ATP in a biological context.

17 For error-correcting codes, a theoretical limit on the necessary redundancy to be able to correct errror was discovered long ago by Shannon (1948), but has proven impossible to reach practically—the reason is that the correction problem is frustrated, as well as effectively disordered, and the Shannon limit is the analogue of a thermodynamical transition, while the algorithms trying to reach it practically are dynamical with consequent glassy hindrance.

18 There are typically no direct interactions, but, as noted earlier, individual interaction with a high-dimensional and varying information field provides an effective inter-agent interaction with *α*=*N*/2.

19 For example, in the MG, increasing the number of players while maintaining the information dimension can lead to a transition through the critical *α* separating ergodic and non-ergodic regimes, thereby providing a natural limit on the usefulness of entering the fray.
